# Age patterns of nonalcoholic fatty liver disease incidence: heterogeneous associations with metabolic changes

**DOI:** 10.1186/s13098-022-00930-w

**Published:** 2022-11-28

**Authors:** Yansong Lin, Xiongcai Feng, Xu Cao, Rong Miao, Yanhong Sun, Rui Li, Junzhao Ye, Bihui Zhong

**Affiliations:** 1 Department of Gastroenterology, The First Affiliated Hospital, Sun Yat-Sen University, No. 58 Zhongshan II Road, Yuexiu District, Guangzhou, 510080 China; 2 Physical Examination Center, The East Division of the First Affiliated Hospital, Sun Yat-Sen University, Guangzhou, 510700 Guangdong China; 3 Department of Clinical Laboratory, The First Affiliated Hospital of Sun Yat-Sen University, Guangzhou, China

**Keywords:** Nonalcoholic fatty liver disease, Incidence rate, Aging, Risk factors, Metabolic disorder

## Abstract

**Background:**

As the nonalcoholic fatty liver disease (NAFLD) epidemic matures, understanding how metabolic changes in NAFLD development vary over the age distribution is important to guide precise prevention. We aimed to clarify metabolic trends in age-specific NAFLD incidence.

**Methods:**

We conducted a 4-year longitudinal retrospective cohort study enrolling 10,240 consecutive healthy individuals who received annual physical examination during 2012–2019. Baseline and dynamic changes in metabolism and hepatic steatosis determined with ultrasound were collected and analyzed stratified by age into the following groups: 20–34, 35–49, 50–64, and over 65 years.

**Results:**

Overall, 1701 incident NAFLD participants (16.6%) were identified. Adjusted Cox regression analysis showed that the baseline and increased body mass index were the main risk factors for NAFLD in people ≤ 65 years old. Baseline high-density lipoprotein (HR = 0.56; 95% CI 0.39–0.78) was a protective factor for newly onset NAFLD in the 50-to-64-year-old group, while baseline SBP (HR = 1.03; 95% CI 1.01–1.05), baseline uric acid (HR = 1.04; 95% CI 1.01–1.07), triglyceride increase (HR = 4.76; 95% CI 3.69–6.14), fasting blood glucose increase (HR = 1.32; 95% CI 1.06–1.65) were independently associated with incident NAFLD in over-65-year-old group.

**Conclusions:**

NAFLD incidence attributable to potentially metabolic risk factors varied substantially across age groups in a cohort of Chinese people. The adoption of age targeted metabolic prevention strategies might reduce the burden of NAFLD.

**Supplementary Information:**

The online version contains supplementary material available at 10.1186/s13098-022-00930-w.

## Introduction

Nonalcoholic fatty liver disease (NAFLD), which is characterized by excessive fat deposition in the hepatic parenchyma is the fastest rising etiology of chronic liver disease in both developed and developing countries [1, 2]. NAFLD has experienced a dramatic increase in the last 20 years, with the global prevalence estimated to be 26%. In China, its prevalence was reported to be as high as 29.81% in 2019 [3]. Furthermore, NAFLD not only has reciprocal relationship with metabolic disorders including obesity, hypertension, dyslipidemia, diabetes, gout, and atherosclerotic diseases, but can also progress to liver fibrosis, cirrhosis and liver cancer due to a steatosis-inflammatory-fibrosis response, causing a dramatic burden to patients and society [4]. Given its increasing prevalence, identifying disparities in the metabolic risk factors of NAFLD is of great significance to effectively control the disease.

Aging and metabolic abnormalities have been revealed as the major important independent factors related to the progression of NAFLD across different predictive models [5]. However, aging intrinsically induces complex changes in systemic metabolism, including changes in the glucose, lipid, amino acid, and nucleotide metabolism [6]. A recent study demonstrated that the energy expenditure related to metabolism in humans from the age of 1 to 95 did not decreased until the age of 60, and by the age of 95, it was only 74% of that of middle-aged people [7]. The research suggested that the role of the decrease in metabolic rate with increasing age in metabolic disorders remained inconclusive. Therefore, the relationship between serum metabolic signature dynamic changes, aging and fatty liver remains to be unraveled.

China was considered a nonepidemic area in the past [8], however according to a Markov model based on the risk factor data of NAFLD, China is predicted to have the largest growth rate of NAFLD in the world, with a higher rate in the economically developed regions [9–14]. The aim of this study was to analyze the incidence rate and metabolic profile changes of NAFLD in distinct age groups among physical examination population recruited from Guangzhou city, a high-epidemic area of NAFLD in China.

## Methods

### Study design and population

This study was a retrospective population-based cohort study approved by the ethics committee of the First Affiliated Hospital of Sun Yat-sen University (ethics code [2020] No. 187). A total of 10,240 people who underwent annual health check-ups and had no NAFLD at baseline in the First Affiliated Hospital of Sun Yat-sen University during January 2012 to December 2019 were recruited. Inclusion criteria: participants older than 20 years old with complete data of anthropometric, laboratory and abdominal ultrasound. Exclusion criteria: participants with existing with known liver diseases (including fatty liver, viral hepatitis, autoimmune liver disease, liver cirrhosis, hepatocellular carcinoma, etc.); Excessive alcohol consumption (alcohol intake ≥ 30 g/day for men and ≥ 20 g/day for women); Medication of hyperlipidemia, hyperglycemic, hypertension or hyperuricemia; Pregnancy.

### Clinical and laboratory data collection

Each participant was required to complete a questionnaire for self-reported alcohol consumption. The alcohol consumption was evaluated by an experienced physician. The drinking history including the following items: (1) drink or not; (2) if drink, the type of the drink; (3) the consumption of drink per week; (4) have ever given up drinking or not. The physical examination reports, including anthropometric, laboratory and imaging examination results, were extracted through the electronic medical record system, and information about physical examination time, age, gender, medical history, personal history, and family history of all the enrolled were also recorded. Current smoker was classified as those with a history of smoking in previous 1 year. Anthropometric indexes were measured including height, weight, body mass index (BMI), systolic blood pressure (SBP), diastolic blood pressure (DBP). Laboratory indexes comprised of the following blood indicators: alanine aminotransferase (ALT), aspartate aminotransferase (AST), γ-Glutamyl transpeptidase (GGT), total cholesterol (CHOL), triglycerides (TG), high-density lipoprotein cholesterol (HDL-c), low-density lipoprotein cholesterol (LDL-c), fasting blood glucose (FBG), creatinine (Cr), blood urea nitrogen (BUN), uric acid (UA). Noninvasive fibrosis scores, including the fibrosis index based on the 4 factors (FIB-4) and AST to platelet ratio index (APRI), were computed from serological examination data according to the published algorithms [15]. The presence of advance fibrosis was defined as: FIB-4 > 2.67, and APRI > 0.7 [15, 16]. The patients were screened for viral hepatitis by blood tests of hepatitis B surface antigen and antibody against hepatitis C virus.

### Ultrasonography

The criteria for the ultrasonic diagnosis of fatty liver were established according to the Chinese guidelines for the prevention and treatment of nonalcoholic fatty liver disease (updated in 2018) [17], and include the following conditions: the characteristics of liver anterior field echo enhancement (“bright liver”), far-field echo attenuation and unclear display of intrahepatic tubular structure. Ultrasonography examination was performed by two physicians with over 5 years of experience in ultrasound measurement using high-resolution B-mode ultrasonography with Mylab 70 and Mylab 90 scanners (Esaote SpA, Genoa, Italy).

### Definition of newly onset metabolic risk comorbidities

ALT exceeding the upper limit of normal value, which is 30 U/L for men and 19 U/L for women, or the GGT levels exceeding 50 U/L, were both defined as elevated liver enzymes [18]. Newly onset hypertension was defined as the an SBP from < 130 mmHg to ≥ 130 mmHg, DBP from < 85 mmHg to ≥ 85 mmHg or specific drug treatment compared to the previous year. Newly onset glucose abnormality was defined as fasting glucose levels from < 5.6 mmol/L to ≥ 5.6 mmol/L or a change from prediabetic levels [fasting glucose levels 5.6 to 6.9 mmol/L] to levels characteristic of diabetes mellitus [fasting glucose levels ≥ 7.0 mmol/L] compared to the previous year. Newly onset hyperlipidemia was defined as plasma triglycerides from < 1.70 mmol/L to ≥ 1.70 mmol/L, plasma HDL-cholesterol from ≥ 1.0 mmol/L to < 1.0 mmol/L for men and from ≥ 1.3 mmol/L to < 1.3 mmol/L for women or specific drug treatment compared to the previous year. Newly onset hyperuricemia was defined as plasma uric acid from ≤ 430 μmol/L to > 430 μmol/L for men and from ≤ 360 μmol/L to > 360 μmol/L for women or specific drug treatment compared to the previous year [19].

### Follow-up

The follow-up assessments were conducted annually during the observation period. The evaluation parameters for follow-up, including anthropometric, laboratory and ultrasonography indicators for follow-up were the same as those measured at baseline. For patients who developed steatosis during the follow-up, the date when fatty liver was detected for the first time was considered the last follow-up, while those who did not develop fatty liver were assessed until the fifth annual evaluation.

### Statistical analysis

SPSS (version 25.0) was applied for data analysis. Continuous variables are expressed as the mean ± standard deviation or median with IQR. Two groups of continuous variables were compared by independent sample t test, and multiple groups were compared by ANOVA test. Bonferroni correction was adopted for intergroup comparison. The Kruskal Wallis rank sum test was used for measurement data, and Pearson chi square test was used for frequency comparison of counting data. Cox regression was used to analyze the risk factors related to the incidence of NAFLD. The trends in longitudinal analyses were analyzed using generalized additive models using smooth functions (i.e., splines) to describe possible non-linear relationships [20]. The predicting value of the models was estimated by the area under the receiver operating characteristic curve. A two-sided test of P < 0.05 was defined as statistically significant.

## Results

### Clinical characteristics of subjects with newly onset NAFLD during follow-up

**Fig. 1:**
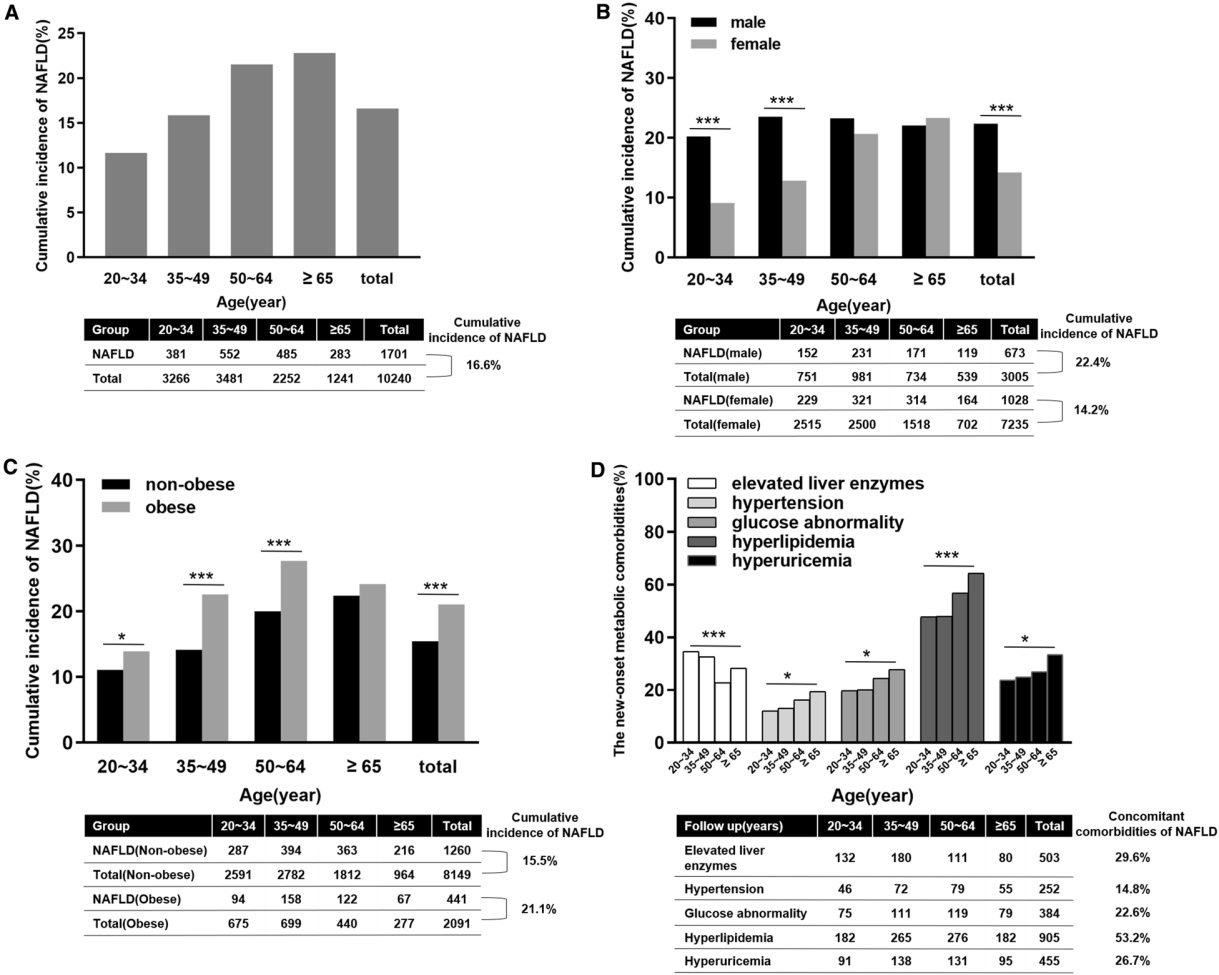
The incidence of NAFLD in different age (**A**), gender (**B**) and obesity status (**C**), as well as the ratio of new-onset metabolic abnormalities along with incident NAFLD (**D**) during follow up. (*p < 0.05; **p < 0.01; ***p < 0.001)

We enrolled 10,240 subjects without NAFLD at baseline (Additional file 1: Fig. S1). The baseline characteristics of the included and excluded subjects did not show significant differences (Additional file 1: Table S1). After 4 years of follow-up, 1701 of 10,240 subjects without NAFLD at baseline developed NAFLD, with an overall incidence rate of 16.6% and an annual incidence rate of 4.15% (Fig. 1A). The proportion of males and the baseline BMI of the newly onset NAFLD group were significantly higher than those in the non-NAFLD development group. Compared with patients who did not develop NAFLD, patients in the newly onset NAFLD group had higher levels of ALT, AST, GGT, CHOL, TG, LDL-c, FBG, Cr and UA. At the end point, the proportion of patients with hypertension, diabetes mellitus, hyperlipidemia, advanced fibrosis, and a high BMI (23.6 ± 2.8 vs. 22.1 ± 2.6 kg/m^2^, P < 0.001) was also higher in the newly onset NAFLD group. Moreover, the levels of ALT, AST, GGT, CHOL, TG, LDL-c, FBG, Cr and UA (P < 0.001) were also higher in this group than in patients who did not develop NAFLD. The differences between the end point and the baseline showed that, the increases in BMI, ALT, AST, CHOL, TG, LDL-c, FBG and UA were higher (P < 0.001) in the newly onset NAFLD group than in patients who did not develop NAFLD (Table 1).

**Table 1:** Baseline and follow up data for study population

Characteristic	Baseline			Last follow-up			Change from baseline		
	Non-NAFLD (n = 8539)	NAFLD (n = 1701)	*P*	Non-NAFLD (n = 8539)	NAFLD (n = 1701)	*P*	Non-NAFLD (n = 8539)	NAFLD (n = 1701)	*P*
Male (n, %)	2332 (27.3)	673 (39.6)	< 0.001	–	–	–	–	–	–
Age (year)	43.0 ± 15.1	48.0 ± 15.4	< 0.001	47.0 ± 15.1	51.3 ± 15.8	< 0.001	–	–	–
Current smoker, n (%)	1836 (21.5)	378 (22.2)	0.510	2015 (23.6)	417 (24.5)	0.417	–	–	–
Hypertension, n (%)	1716 (20.1)	359 (21.1)	0.344	1998 (23.4)	512 (30.1)	< 0.001	–	–	–
Diabetes mellitus, n(%)	1067 (12.5)	225 (13.2)	0.406	1324 (15.5)	437 (25.7)	< 0.001	–	–	–
Hyperlipidemia, n (%)	1093 (12.2)	282 (16.6)	< 0.001	1298 (15.2)	1187 (69.8)	< 0.001	–	–	–
FIB-4 ≥ 2.67	248 (2.9)	51 (3.0)	0.834	273 (3.2)	83 (4.9)	0.001	–	–	–
APRI ≥ 0.7	222 (2.6)	46 (2.7)	0.805	256 (3.0)	75 (4.4)	0.003	–	–	–
Weight (kg)	59.5 ± 9.3	61.0 ± 9.1	< 0.001	59.4 ± 9.8	64.0 ± 9.9	< 0.001	− 0.1 ± 2.8	3.1 ± 2.7	< 0.001
BMI (kg/m^2^)	22.1 ± 2.4	22.5 ± 2.5	< 0.001	22.1 ± 2.6	23.6 ± 2.8	< 0.001	− 0.03 ± 1.0	1.1 ± 1.0	< 0.001
SBP (mmHg)	116 ± 12	118 ± 12	< 0.001	119 ± 12	120 ± 13	0.059	2 ± 10	1 ± 11	0.250
DBP (mmHg)	74 ± 7	75 ± 7	< 0.001	78 ± 8	77 ± 8	< 0.001	3 ± 6	2 ± 7	< 0.001
ALT (U/L)	16 (13–23)	19 (14–26)	< 0.001	17 (13–24)	23 (17–33)	< 0.001	0 (− 4 to 5)	4 (− 3 to 12)	< 0.001
AST (U/L)	20 (17–25)	21 (18–25)	< 0.001	21 (18–26)	24 (20–29)	< 0.001	1 (− 3 to 5)	3 (− 1 to 7)	< 0.001
GGT (U/L)	20 (16–24)	20 (16–23)	0.004	24 (19–29)	23 (18–28)	< 0.001	4 (− 3 to 10)	3 (− 3 to 10)	0.091
LDH (U/L)	163 (139–188)	162 (139–186)	0.173	168 (141–195)	165 (139–191)	0.004	6 (− 29 to 37)	3 (− 26 to 35)	0.208
CHOL (mmol/L)	5.0 ± 0.9	5.3 ± 0.9	< 0.001	5.0 ± 0.9	6.2 ± 1.2	< 0.001	− 0.2 ± 0.9	0.9 ± 1.2	< 0.001
TG (mmol/L)	1.00 ± 0.45	1.36 ± 0.59	< 0.001	1.05 ± 0.42	2.31 ± 0.89	< 0.001	− 0.01 ± 0.50	1.17 ± 1.62	< 0.001
HDL-c (mmol/L)	1.48 ± 0.39	1.42 ± 0.36	< 0.001	1.52 ± 0.37	1.38 ± 0.43	< 0.001	-0.02 ± 0.63	− 0.14 ± 0.62	< 0.001
LDL-c (mmol/L)	3.17 ± 0.74	3.44 ± 0.876	< 0.001	3.18 ± 0.73	3.86 ± 1.01	< 0.001	0.28 ± 1.38	0.68 ± 1.51	< 0.001
FBG (mmol/L)	5.2 ± 0.8	5.3 ± 0.9	< 0.001	5.2 ± 0.6	5.6 ± 0.8	< 0.001	− 0.1 ± 1.1	0.3 ± 1.2	< 0.001
Creatinine (μmol/L)	67.2 ± 11.6	71.7 ± 16.7	< 0.001	66.7 ± 11.1	72.4 ± 13.1	< 0.001	− 0.4 ± 15.8	0.6 ± 17.8	0.169
BUN (mmol/L)	5.6 ± 1.4	5.6 ± 1.4	0.514	5.8 ± 1.0	5.8 ± 1.1	0.875	0.1 ± 2.1	0.2 ± 2.0	0.250
Uric acid (μmol/L)	301 ± 45	325 ± 50	< 0.001	316 ± 50	369 ± 55	< 0.001	15 ± 40	44 ± 72	< 0.001
Platelet (*10^9^/L)	239 ± 40	243 ± 41	0.046	243 ± 40	245 ± 42	0.027	4 ± 30	2 ± 28	0.301

Data are expressed as n (%), mean ± standard deviation and median (quartile)

Because all patients with completed follow-up for 4 years, therefore gender and difference of ages doesn’t change. Non-NAFLD means that the patients didn’t occur NAFLD during follow-up; NAFLD means that the patients occurred NAFLD during follow-up

*NAFLD* non-alcoholic fatty liver disease, *FIB-4* fibrosis index based on the 4 factors, *APRI* AST-to-platelet ratio index, *BMI* body mass index, *SBP* systolic blood pressure, *DBP* diastolic blood pressure, *ALT* alanine aminotransferase, *AST* aspartate aminotransferase, *GGT* gamma glutamyl transpeptidase, *LDH* lactic dehydrogenase, *CHOL* total cholesterol, *TG* triglycerides, *HDL-c* high-density lipoprotein-cholesterol, *LDL-c* low-density lipoprotein-cholesterol, *FBG* fasting blood glucose, *BUN* blood urea nitrogen

The incidence rate of NAFLD increased in a stepwise manner with age, 11.7%, 15.9%, 21.5% and 22.8% in the 20–34, 35–49, 50–64 and over-65-year-old groups, respectively (P < 0.001). The incidence of NAFLD was highest in the 35–49-year-old group in men (23.5%) and in over-65-years-old group in women (23.4%) (Fig. 1B). The incidence rate of NAFLD in obese people in 20–34, 35–49, 50–64-year-old groups were higher than that in nonobese subjects (P < 0.05) (Fig. 1C). The rates of concomitant elevated liver enzymes (34.6% vs. 32.6% vs. 22.9% vs. 28.3%, P < 0.001), hypertension (12.1% vs. 13.0% vs. 16.3% vs. 19.4%, P = 0.026), glucose abnormalities (19.7% vs. 20.1% vs. 24.5% vs. 27.9%, P = 0.024), hyperlipidemia (47.8% vs. 48.0% vs. 56.9% vs. 64.3%, P < 0.001) and hyperuricemia (23.9% vs. 25.0% vs. 27.0% vs. 33.6%, P = 0.027) were significantly different among the four groups (Fig. 1D). Comparing the clinical characteristics of different age groups at baseline showing that the BMI (22.5 ± 2.6 kg/m^2^) in the 35–49-year-old group was the highest, while the blood pressure was the highest in the over-65-year-old group (SBP: 122 ± 13 mmHg, DBP: 79 ± 7 mmHg). In terms of metabolic indices, the CHOL level (5.6 ± 0.8 mmol/L) and LDL-c level (3.66 ± 0.72 mmol/L) were highest in the 50–64-year-old-group, while the levels of TG (1.47 ± 0.60 mmol/L), FBG (5.8 ± 1.3 mmol/L), creatinine(76 ± 17 μmol/L) and UA (342 ± 53 μmol/L)were highest in the over-65-year-old group (Additional file 1: Table S2).

### Incidence rate of NAFLD stratified by metabolic indicators and age

**Fig. 2:**
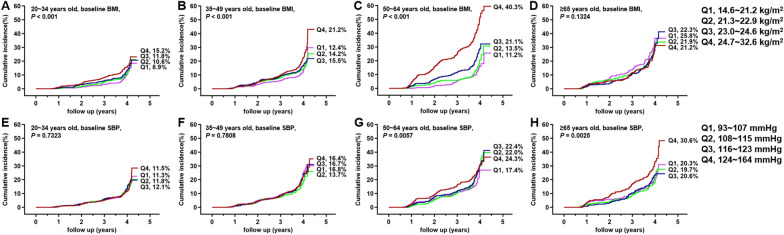
The cumulative incidence of NAFLD in the quartiles (Q) of baseline risk factors for the new-onset of NAFLD. **A**–**D** Baseline BMI; **E**–**H** baseline SBP. The corresponding quartiles of the variables were shown on the right side of the picture. Because all patients with completed follow-up for 4 years, therefore the numbers of patients at risk for each K–M curves were not shown

**Fig. 3:**
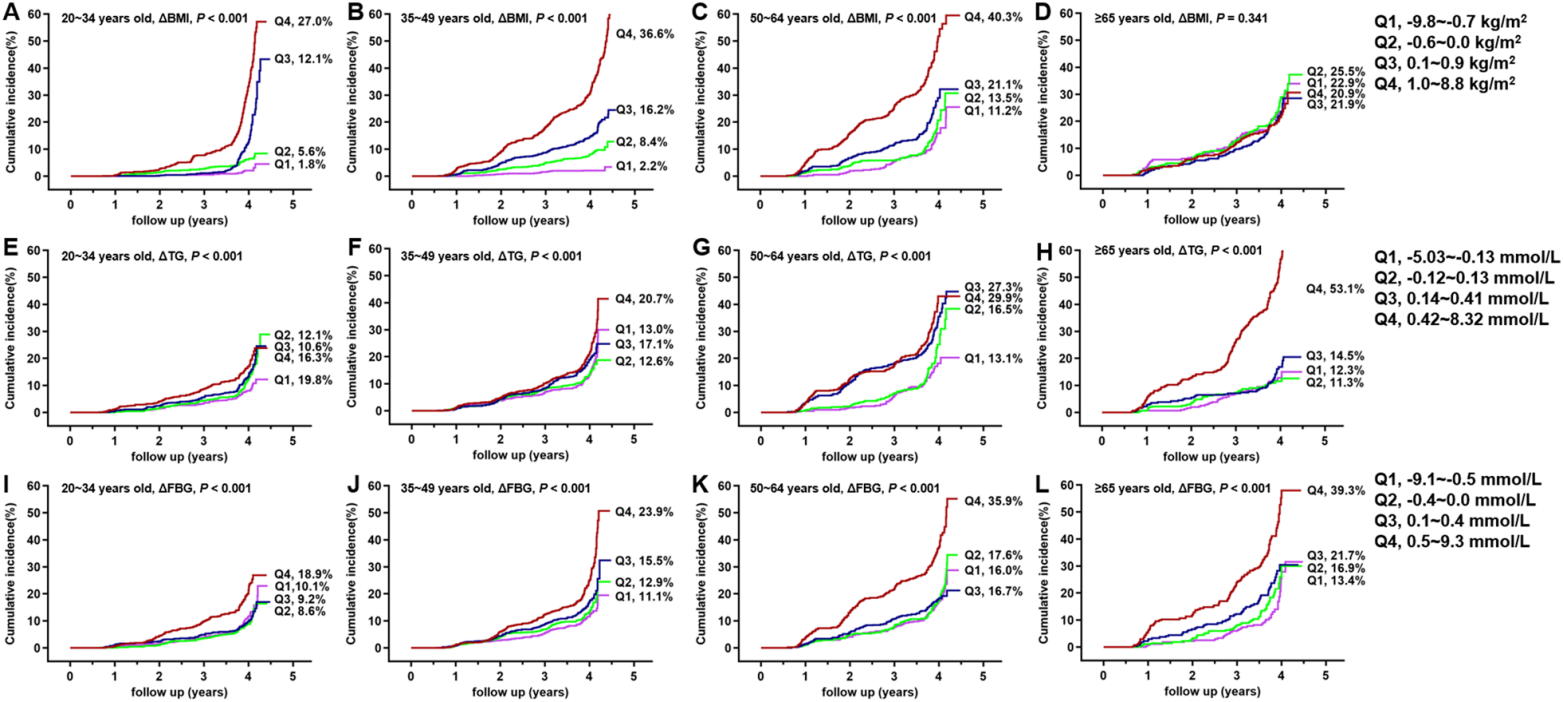
The cumulative incidence of NAFLD in the quartiles (Q) of dynamic changes of risk factors for the new-onset of NAFLD. **A**–**D** ΔBMI; **E**–**H** ΔTG; **I**–**L** ΔFBG. The corresponding quartiles of the variables were shown on the right side of the picture. Because all patients with completed follow-up for 4 years, therefore the numbers of patients at risk for each K–M curves were not shown

**Fig. 4:**
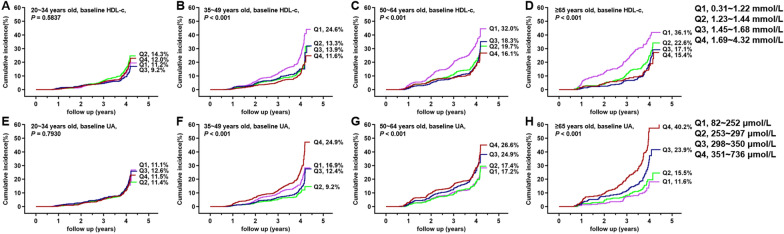
The cumulative incidence of NAFLD in the quartiles (Q) of baseline risk factors for the new-onset of NAFLD. **A**–**D** Baseline HDL-c; **E**–**H** baseline UA. The corresponding quartiles of the variables were shown on the right side of the picture. Because all patients with completed follow-up for 4 years, therefore the numbers of patients at risk for each K–M curves were not shown

Variations in the risk factors for the incidence of NAFLD during follow-up are shown in Additional file 1: Fig. S2. The levels of BMI gradually increased in people aged 20–49 years old but remained stable in people aged ≥ 50 years old. The levels of TG and UA, they gradually increased in people aged ≥ 50 years old but remained stable in people aged 20–49 years old. The levels of SBP, HDL-c and FBG were relatively stable in specific group during follow-up. The relationship between the above risk factors and the cumulative incidence rate of NAFLD was analyzed further. In the 20–34-year-old group, the cumulative incidence rate of NAFLD increased as the quantile of the BMI change value increased, but increases in the baseline BMI quantile did not correlate with this incidence rate (Figs. 2A, 3A, P < 0.001). In the 35–49-years-old group, the cumulative incidence rate of NAFLD increased gradually with the quantile increases in BMI, HDL-c, UA at baseline and change in BMI, TG and FBG (Figs. 2B, 3B, F, J, 4B, F; P < 0.05). For patients older than 65 years, the cumulative incidence rate of NAFLD increased with increasing metabolic indices, including the baseline levels of SBP, and UA, changes in the of TG and FBG and the decreases in the baseline level of HDL-c (Figs. 2H, 3H, L, 4D, H; P < 0.05). The quantile of the baseline creatinine and change in creatinine were not significant associated with the cumulative incidence rate of NAFLD stratified by age (Additional file 1: Fig. S3).

### Risk factors for newly onset NAFLD among varied age groups

**Fig. 5:**
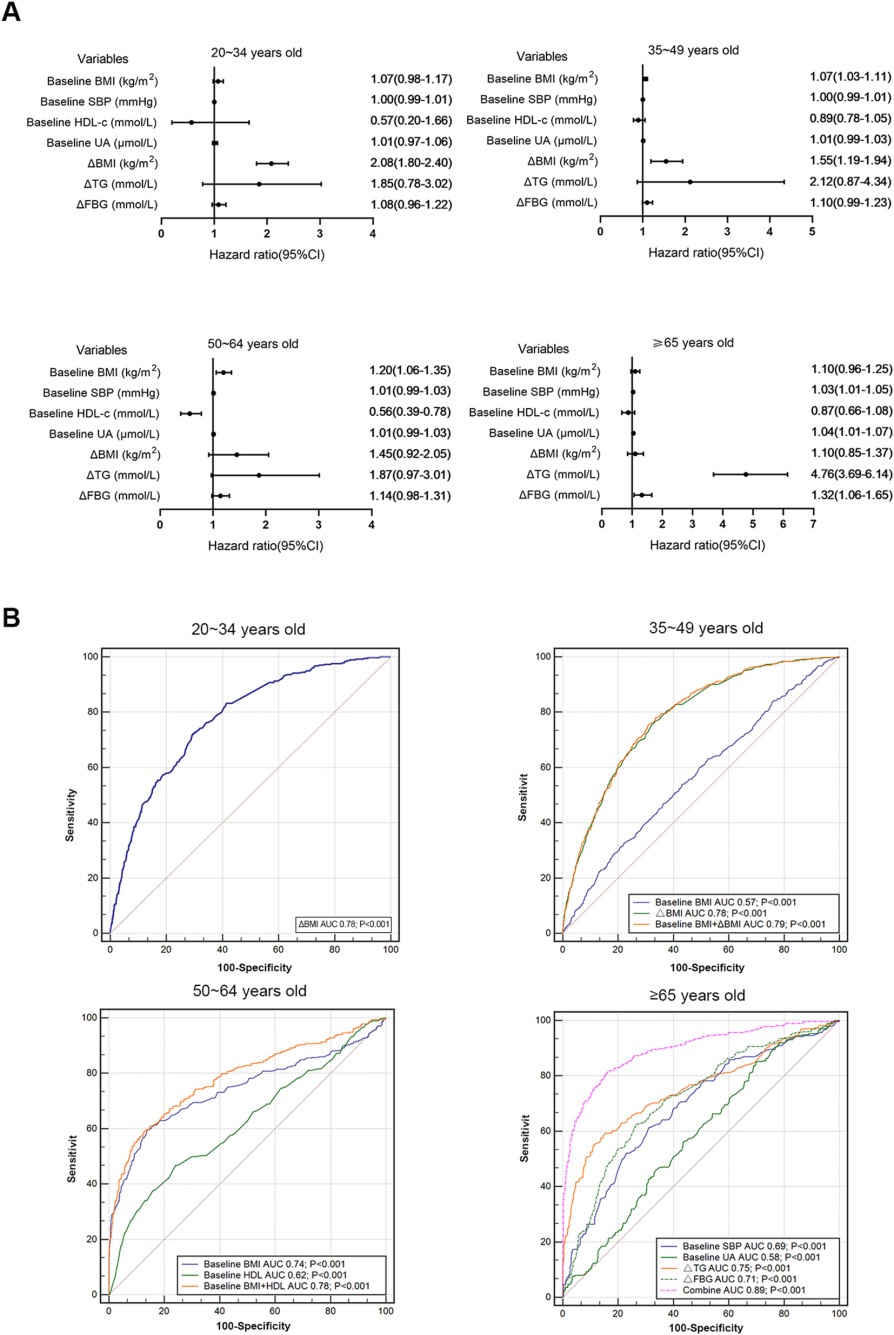
Forest plot of the risk factors for incidence of NAFLD (**A**) and receiver operator characteristic (ROC) curve of factors that predict incidence of NAFLD in different groups (**B**)

Univariate Cox regression analysis showed that the levels of baseline BMI, HDL-c, LDL-c, and FBG and changes in the BMI, TG, HDL-c, FBG, and UA were risk factors of the new-onset of NAFLD in the general population. After multivariate adjustment, changes in the BMI, TG, HDL-c, FBG, and UA, as well as the level of baseline FBG remained independently associated with newly onset of NAFLD. After stratification into subgroups according to age, the change in BMI (HR = 2.08, 95% CI 1.80–2.40, P < 0.001) was the only independent risk factor for new onset of NAFLD in 20–34-years-old group. Baseline BMI (HR = 1.07, 95% CI 1.03–1.11) and the change in BMI (HR = 1.55, 95% CI 1.19–1.94) were independently associated with new onset of NAFLD in people aged 35–49 years old (all P < 0.001). For the over-65-year-old group, the baseline SBP (HR = 1.03, 95% CI 1.01–1.05), UA level (HR = 1.04, 95% CI 1.01–1.07), the change in TG (HR = 4.76, 95% CI 3.69–6.14), and FBG (HR = 1.32, 95% CI 1.06–1.65) were all risk factors for NAFLD development (Additional file 1: Table S3, Table 2, Fig. 5A).

**Table 2:** The multivariable Cox regression analysis of risk factors for incidence of NAFLD during follow-up

Variable	ALL	*P*	20–34 years old	*P*	35–49 years old	*P*	50–64 years old	*P*	≥ 65 years old	*P*
	HR (95%CI)		HR (95%CI)		HR (95%CI)		HR (95%CI)		HR (95%CI)	
Male	–	–	–	–	1.50 (1.20–1.82)	< 0.001	–	–	1.41 (0.88–1.98)	0.54
Baseline factors										
BMI (increase 1 kg/m^2^)	1.08 (0.96–1.20)	0.20	–	–	1.07 (1.03–1.11)	< 0.001	1.20 (1.06–1.35)	< 0.001	–	–
SBP (increase 1 mmHg)	–	–	–	–	–	–	1.01 (0.99–1.03)	0.22	1.03 (1.01–1.05)	< 0.001
DBP (increase 1 mmHg)	–	–	–	–	–	–	–	–	–	–
ALT (increase 1U/L)	–	–	1.00 (0.99–1.02)	0.23	1.00 (0.99–1.02)	0.21	–	–	1.00 (0.99–1.01)	0.25
AST (increase 1U/L)	–	–	1.01 (0.98–1.04)	0.33	1.01 (0.98–1.04)	0.42	–	–	–	–
GGT (increase 1U/L)	–	–	–	–	0.99 (0.96–1.02)	0.12	–	–	–	–
LDH (increase 1U/L)	–	–	–	–	–	–	–	–	–	–
CHOL (increase 1 mmol/L)	–	–	1.18 (0.96–1.42)	0.32	1.20 (0.94–1.50)	0.45	1.21 (0.97–1.60)	0.34	1.32 (0.96–1.72)	0.23
TG (increase 1 mmol/L)	–	–	–	–	–	–	1.32 (0.98–1.75)	0.08	1.50 (0.80–2.35)	0.56
HDL-c (increase 1 mmol/L)	0.90 (0.71–1.11)	0.20	–	–	–	–	0.56 (0.39–0.78)	< 0.001	–	–
LDL-c (increase 1 mmol/L)	1.25 (1.08–1.45)	0.003	–	–	1.25 (0.85–1.70)	0.22	1.16 (0.95–1.45)	0.15	1.29 (0.88–1.85)	0.20
FBG (increase 1 mmol/L)	1.22 (1.10–1.36)	< 0.001	–	–	–	–	–	–	–	–
Creatinine (increase 1 μmol/L)	–	–	–	–	1.00 (0.99–1.01)	0.77	–	–	–	–
BUN (increase 1 mmol/L)	–	–	–	–	–	–	–	–	–	–
Uric acid (increase 1 μmol/L)	–	–	–	–	1.01 (0.99–1.03)	0.12	–	–	1.04 (1.01–1.07)	< 0.001
Dynamic factors										
ΔBMI (increase 1 kg/m^2^)	1.66 (1.52–1.82)	< 0.001	2.08 (1.80–2.40)	< 0.001	1.55 (1.19–1.94)	< 0.001	1.45 (0.92–2.05)	0.45	–	–
ΔSBP (increase 1 mmHg)	–	–	–	–	–	–	–	–	–	–
ΔDBP (increase 1 mmHg)	–	–	1.00 (0.99–1.01)	0.34	1.01 (0.99–1.03)	0.13	–	–	–	–
ΔALT (increase 1U/L)	–	–	1.00 (0.99–1.02)	0.41	1.01 (0.98–1.04)	0.35	1.01 (0.99–1.03)	0.10	–	–
ΔAST (increase 1U/L)	–	–	1.01 (0.98–1.04)	0.61	1.00 (0.99–1.01)	0.44	1.01 (0.99–1.02)	0.12	1.01 (0.99–1.03)	0.23
ΔGGT (increase 1U/L)	1.01 (0.99–1.03)	0.21	–	–	–	–	1.01 (0.99–1.03)	0.21	–	–
ΔLDH (increase 1U/L)	–	–	–	–	–	–	–	–	–	–
ΔCHOL (increase 1 mmol/L)	–	–	1.08 (0.96–1.22)	0.40	1.16 (0.98–1.36)	0.12	1.11 (0.87–1.30)	0.25	1.43 (0.97–2.02)	0.09
ΔTG (increase 1 mmol/L)	1.86 (1.28–2.56)	< 0.001	–	–	–	–	1.87 (0.97–3.01)	0.10	4.76 (3.69–6.14)	< 0.001
ΔHDL-c (increase 1 mmol/L)	0.60 (0.51–0.69)	< 0.001	–	–	–	–	–	–	–	–
ΔLDL-c (increase 1 mmol/L)	–	–	1.23 (0.98–1.56)	0.08	1.08 (0.98–1.20)	0.10	1.18 (0.90–1.40)	0.43	1.15 (0.97–1.38)	0.23
ΔFBG (increase 1 mmol/L)	1.11 (1.02–1.21)	0.014	1.08 (0.96–1.22)	0.10	1.10 (0.99–1.23)	0.07	1.14 (0.98–1.31)	0.20	1.32 (1.06–1.65)	< 0.001
ΔCreatinine (increase 1 μmol/L)	–	–	–	–	–	–	–	–	–	–
ΔBUN (increase 1 mmol/L)	–	–	–	–	–	–	–	–	–	–
ΔUric acid (increase 1 μmol/L)	1.01 (1.00–1.02)	< 0.001	–	–	1.01 (0.99–1.03)	0.32	1.02 (0.98–1.04)	0.32	1.01 (0.99–1.03)	0.12

Following are the adjustment variables included in multivariate analysis. ALL: age, sex, BMI, HDL-c, LDL-c, FBG, ΔBMI, ΔTG, ΔHDL-c, ΔFBG, ΔUric acid; 20–34 years old: sex, ALT, AST, CHOL, ΔBMI, ΔALT, ΔAST, ΔCHOL, ΔLDL-c, ΔFBG; 35–49 years old: sex, BMI, ALT, AST, GGT, CHOL, LDL-c, Creatinine, Uric acid, ΔBMI, ΔDBP, ΔALT, ΔAST, ΔCHOL, ΔLDL-c, ΔFBG, ΔUric acid; 50–64 years old: sex, BMI, SBP, CHOL, TG, HDL-c, LDL-c, ΔBMI, ΔALT, ΔAST, ΔGGT, ΔCHOL, ΔTG, ΔLDL-c, ΔFBG, ΔUric acid; ≥ 65 years old: sex, SBP, ALT, CHOL, TG, LDL-c, Uric acid, ΔAST, ΔCHOL, ΔTG, ΔLDL-c, ΔFBG, ΔUric acid

Δ = change in variable i.e. follow-up minor bsaeline measurement

*NAFLD* non-alcoholic fatty liver disease, *HR* hazard ratio, *BMI* body mass index, *SBP* systolic blood pressure, *DBP* diastolic blood pressure, *ALT* alanine aminotransferase, *AST* aspartate aminotransferase, *GGT* gamma glutamyl transpeptidase, *LDH* lactic dehydrogenase, *CHOL* total cholesterol, *TG* triglycerides, *HDL-c* high-density lipoprotein-cholesterol, *LDL-c* low-density lipoprotein-cholesterol, *FBG* fasting blood glucose, *BUN* blood urea nitrogen

### Individualized prediction accuracy of NAFLD based on age-related risk factors

The area under the curve (AUC) of BMI increase for predicting newly onset NAFLD in 20–34-year-old group was 0.78 (P < 0.001). For individuals aged 35–49 years old, the AUCs of the baseline level of BMI and increased BMI were 0.57 and 0.78, respectively (P < 0.001), and the combined of the two increased the diagnostic accuracy (AUC = 0.79, P < 0.001). The AUCs of baseline BMI and baseline HDL-c for people aged 50–64 years old were 0.74 and 0.62, respectively (P < 0.001), and their combination increased diagnostic accuracy (AUC = 0.78, P < 0.001). In the over-65-year-old group, the AUCs of the level of baseline SBP, baseline UA level, increased TG and increased FBG level were 0.69, 0.58, 0.75 and 0.71, respectively (all P < 0.001), and the AUC maximized when combining the four indices (AUC 0.89; P < 0.001) (Fig. 5B).

## Discussion

In the current study, which examined a large sample of over 10 thousand individuals, we have demonstrated that dynamic changes in metabolic profiles contributed to various incidence of NAFLD among healthy individuals in different age subgroups. For people younger than 50 years old, baseline BMI or changes in BMI had a stronger association incident NAFLD, while in people older than 65 years old, metabolic factors other than BMI were more related to NAFLD occurrence. Furthermore, the areas under the ROC curves indicated that significant age related factors were slightly superior to BMI for predicting NAFLD incidence. These heterogenetic associations among age groups with metabolic changes in incident NAFLD may benefit clinicians in screening adults who are at a potentially higher risk for developing NAFLD.

This study included subjects recruited during health check-ups who were free of NAFLD at baseline. The cumulative incidence rate was 16.6%, and the annual incidence rate was 4.15%. Similarly, the cumulative incidence rate of NAFLD reported in Europe and America was 8.9–19.0% after follow-up for 1.2–8 years, while the annual incidence rate was 2.7–8.6% [21]. Based on a recent meta-analysis of incident NAFLD case reports from China, it estimated the NAFLD incidence was estimated to be 5.67% (95% CI 4.74–6.68) [11]. Thus, the incidence of NAFLD found in our data was consistent with those reported other studies for the development of NAFLD. Recently a study reported the worldwide long-term trends in the incidence of nonalcoholic fatty liver disease during 1990–2019. The age-standardized incidence rate (ASIR) rapidly increased as age increased for those younger than 45–49 years, decreased for those aged 45–49 years to 65–69 years, and increased for those aged over 65–69 years. The ASIR of Central Latin America was highest (6.88 per 100,000 persons) around the world, while most of the people were white race in Central Latin America. The ASIR of East Asia was 2.10 per 100,000 persons [22]. Furthermore, a study reported that in a longitudinal cohort of 6513 Israelite persons, the incidence rate of NAFLD in the people aged < 45 years old and ≥ 45 years old were 5.8 per 100 person-years and 10.3 per 100 person-years, respectively [23]. In our present study, the annual NAFLD incidence rate in the people aged < 50 years old and ≥ 50 years old were 3.5% and 5.5%, respectively. A study summarized epidemiological features of NAFLD from 1999 to 2018 in China, stating that the annual incidences of NAFLD were higher (4.7% [95% CI 4.0–5.5%]) in the population below age 60 than in the population above age 60 (2.4% [95% CI 2.1–2.8%]) [24]. Therefore, The NAFLD incidence rate in different race would differ by age. Further studies were needed to clarify the NAFLD incidence rate in different race and age.

Weight gain, obesity and various metabolic abnormalities are the risk factors for the occurrence and development of NAFLD. These risk factors have been confirmed not only to be related to simple steatosis of the liver, but also to accelerate the progression of nonalcoholic steatohepatitis (NASH), and even lead to NASH-related cirrhosis and liver cancer [25]. A large-scale epidemiological study in China reported that weight gain in adults increased with age, and this increase was highest at age 45–50 years old then decreased thereafter [26]. In this study, an increase in BMI was a risk factor for newly onset of NAFLD in people younger than 50 years old, which may attributable to the weight gain pattern of Chinese adults, as the previous reported. The weight of people aged 35–64 tended to be stable, and their baseline BMI may be a risk factor for NAFLD. In our study, the baseline BMI was an independent risk factor for NAFLD in the age groups of 20–34 and 35–49 years old. A cohort study of 6310 physical examinees who were followed up for 7 years, found that baseline BMI was a risk factor for NAFLD, and was as an independent risk factor incorporated into a model for predicting the risk of NAFLD [27]. However, the study did not perform a subgroup analysis divided by age. Another study in China of 563 physical examinees with a median age of 59, showed that baseline BMI, SBP, ALT, and increasing BMI were independent risk factors for NAFLD, which is in accordance with the results in this study [28]. BMI is a simple and acknowledged marker that reflects the severity of human obesity, and this study confirmed BMI to be the most powerful factor predicting the risk of NAFLD development; therefore, monitoring BMI in those younger than 60 years would be valuable.

Notably, BMI was not the primary predictor of newly onset NAFLD in individuals older than 65. Our study also found that the baseline SBP and UA as well as increases in the TG and FBG were risk factors for newly onset NAFLD for individuals aged ≥ 65 years. Hypertension is one of the important components of metabolic syndrome, especially in the elderly people. NAFLD is reportedly a risk factor for hypertension, the mechanism of which may mediated by systemic inflammation, insulin resistance and oxidative stress [29]. However, hypertension may also be a new risk factor for NAFLD in return. A large cohort study of 8489 physical examinees without NAFLD or hypertension at baseline found that the development of hypertension was an independent risk factor for newly onset NAFLD after 5 years of follow-up (OR = 1.60, 95% CI 1.30–1.96; P < 0.001) [30]. A large meta-analysis demonstrated that NAFLD is associated with an ~ 1.6-fold increased risk of developing hypertension [31]. Wang followed up 4274 middle-aged and elderly participants for 4.4 years, and supported that the increase in FBG was associated with the onset of NAFLD [32]. In another study of the relationship between UA and NAFLD, Wei, et al. included 2832 non-NAFLD participants who were aged over 63 years or old and found that the cumulative incidence rate of NAFLD increased with increasing baseline serum UA levels after 4 years of follow-up, which clarified that serum UA levels were an independent risk factor for NAFLD in elderly people [33].

Most studies on the incidence of NAFLD often attach much importance to baseline indicators and may ignore the dynamic changes in these indices before the development of NAFLD. Clarifying the relationship between the change in metabolic indicators during the natural course of NAFLD is of great importance for early screening and preventing NAFLD, especially for individuals receiving regular health check-ups. A Japanese study included 27,064 nonobese and non-NAFLD subjects, and found that the risk of NAFLD increased by 60% when weight increased by 3 kg [34]. Rim, et al. studied the relationship between the changes in various indices and the incidence of NAFLD and they found that TG decreases by 9.46 mg/dl in men and 5.98 mg/dl in women as well as a weight increase by 0.36 kg were associated with an increased incidence of NAFLD [35]. However, these studies did not analyze potential differences in risk by age group. By analyzing the ROC curve of risk factors for NAFLD identified from the Cox regression model, we further confirmed the different risk factors of NAFLD in all age groups. In the population aged 20–34, an increased BMI accurately predicted the occurrence of NAFLD, suggesting weight gain should be a focus in the prevention of NAFLD in young people. For people aged 35–49 years old, the AUC of the combination of baseline and increased BMI in predicting NAFLD reached 0.79, suggesting that to prevent NAFLD in middle-aged people, we should not only pay attention to the current body weight but also focus on its dynamic change. In individuals aged 50–64 years old, the AUC of baseline BMI for predicting NAFLD was 0.74, and the prediction accuracy of baseline HDL was low (AUC = 0.62), indicating that weight control is still the primary choice for preventing NAFLD in this stage. For people over 65 years old, combination of baseline SBP, UA, and increases in TG and FBG could accurately predict the onset of NAFLD (AUC = 0.89), suggesting that the management of metabolic abnormalities in the elderly population is particularly important for preventing NAFLD. In conclusion, the risk factors for NAFLD differ by population, and targeted measures should be taken to effectively reduce the incidence of NAFLD.

The shortcomings of this study are as follows: (1) abdominal ultrasound was used to diagnose fatty liver, and mild fatty liver disease may consequently be missed and the severity of fatty liver and fibrosis could not be graded; (2) this study was a retrospective cohort study. Medical history and drinking history data were collected through electronic medical records, which may have some bias; (3) the follow-up time was short, and the conclusions needs further confirmation; (4) This study did not evaluate the impact of lifestyle such as diet and exercise, which may affect the incidence of NAFLD; (5) It was reported that NAFLD has a relationship with the kidney disease endpoint [36]. Since the albuminuria is affected by many factors in this cohort, it may not reflect the relationship between NAFLD and kidney disease progression, so this study did not analyze the data about albuminuria; (6) Recently, the concept of metabolic associated fatty liver disease (MAFLD) was suggested [37]. However, data about waist circumference, ultrasensitive C-reactive protein and fasting insulin were lacking for patients in this study, therefore we didn’t apply the MAFLD concept in this study.

## Conclusions

In summary, the risk factors for NAFLD differ by age group in a cohort of Chinese people. Baseline BMI and changes in the BMI had high predictive value in the young population, whereas metabolism-related factors were more important in the elderly population. In clinical practice, it is necessary to individualize the prevention and treatment of NAFLD need to be individualized according to related risk factors.

## Supplementary Information

**Additional file 1: Table S1.** The baseline characteristics of subjects included and of those excluded. **Table S2.** Baseline characteristic of incident NAFLD patients, group by age. **Table S3.** The univariate Cox regression analysis of risk factors for incidence of NAFLD during follow-up. **Figure S1.** Study flow diagram for longitudinal cohort. **Figure S2.** Metabolic trajectories before NAFLD onset strategies by different ages. **Figure S3.** The cumulative incidence of NAFLD in the quartiles (Q) of baseline creatinine [A–D] and dynamic changes in creatinine [E–H] for newly onset NAFLD. The corresponding quartiles of the variables were shown on the right side of the picture. Because all patients with completed follow-up for 4 years, therefore the numbers of patients at risk for each K–M curves were not shown.

## Data Availability

The datasets used and/or analyzed during the current study are available from the corresponding author upon reasonable request.

## Abbreviations

NAFLD: Nonalcoholic fatty liver disease
BMI: Body mass index
SBP: Systolic blood pressure
DBP: Diastolic blood pressure
ALT: Alanine aminotransferase
AST: Aspartate transaminase GGT: γ-Glutamyl transpeptidase
CHOL: Total cholesterol
TG: Triglycerides
HDL-c: High-density lipoprotein cholesterol
LDL-c: Low-density lipoprotein cholesterol
FBG: Fasting blood glucose
Cr: Creatinine
BUN: Blood urea nitrogen
UA: Uric acid
FIB-4: Fibrosis index based on the 4 factors
APRI: AST to platelet ratio index
AUC: Area under the curve
NASH: Nonalcoholic steatohepatitis
