# Supplementary material for: Age patterns of nonalcoholic fatty liver disease incidence: heterogeneous associations with metabolic changes
Source: Diabetol Metab Syndr. 2022 Nov 28;14:181. doi: 10.1186/s13098-022-00930-w (PMC9706887; doi:10.1186/s13098-022-00930-w)
Supplement: Supplementary file 1 — Additional file 1: Table S1. The baseline characteristics of subjects included and of those excluded. Table S2. Baseline characteristic of incident NAFLD patients, group by age. Table S3. The univariate Cox regression analysis of risk factors for incidence of NAFLD during follow-up. Figure S1. Study flow diagram for longitudinal cohort. Figure S2. Metabolic trajectories before NAFLD onset strategies by different ages. Figure S3. The cumulative incidence of NAFLD in the quartiles (Q) of baseline creatinine [A–D] and dynamic changes in creatinine [E–H] for newly onset NAFLD. The corresponding quartiles of the variables were shown on the right side of the picture. Because all patients with completed follow-up for 4 years, therefore the numbers of patients at risk for each K–M curves were not shown. [file 13098_2022_930_MOESM1_ESM.docx]

Table S1. The baseline characteristics of subjects included and of those excluded.

| **Characteristic** | **Included**  (n=10240) | **Excluded**  (n=17660) | *P* |
| --- | --- | --- | --- |
| Male, n(%) | 3005(29.3) | 5333(30.2) | 0.134 |
| Age (year) | 43.8±15.2 | 44.1±15.0 | 0.109 |
| Weight (kg) | 59.7±9.3 | 59.9±9.4 | 0.086 |
| BMI (kg/m^2^) | 22.2±2.4 | 22.2±2.4 | 0.503 |
| SBP (mmHg) | 116±12 | 116±12 | 0.179 |
| DBP (mmHg) | 74±7 | 74±7 | 0.248 |
| ALT (U/L) | 17(13-23） | 17(12-23) | 0.404 |
| AST (U/L) | 20(17-25) | 20(16-25) | 0.456 |
| GGT (U/L) | 20(16-24) | 20(16-24) | 0.778 |
| LDH (U/L) | 163(139-187) | 164(139-188) | 0.325 |
| CHOL (mmol/L) | 5.0±0.9 | 5.0±0.9 | 0.070 |
| TG (mmol/L) | 1.06±0.48 | 1.07±0.47 | 0.089 |
| HDL-c (mmol/L) | 1.47±0.39 | 1.47±0.39 | 0.302 |
| LDL-c (mmol/L) | 3.21±0.77 | 3.20±0.78 | 0.300 |
| FBG (mmol/L) | 5.2±0.8 | 5.2±0.8 | 0.304 |
| Creatinine (μmol/L) | 67.9±12.7 | 67.7±12.6 | 0.203 |
| BUN (mmol/L) | 5.6±1.4 | 5.6±1.4 | 0.245 |
| Uric acid (μmol/L) | 305±46 | 306±47 | 0.084 |
| Platelet (*10^9^/L) | 240±40 | 241±40 | 0.421 |

Data are expressed as n (%), mean±standard deviation and median (quartile).

Abbreviations: BMI, body mass index; SBP, systolic blood pressure; DBP, diastolic blood pressure; ALT, alanine aminotransferase; AST, aspartate aminotransferase; GGT, gamma glutamyl transpeptidase; LDH, lactic dehydrogenase; CHOL, total cholesterol; TG, triglycerides; HDL-c, high-density lipoprotein-cholesterol; LDL-c, low-density lipoprotein-cholesterol; FBG, fasting blood glucose; BUN, blood urea nitrogen.

Table S2**.** Baseline characteristic of incident NAFLD patients, group by age

| **Characteristic** | **20-34 years old (n=381)** | **35-49 years old (n=552)** | **50-64 years old (n=485)** | **≥65 years old (n=283)** | ***P*** |
| --- | --- | --- | --- | --- | --- |
| NAFLD incidence | 11.7% | 15.9% | 21.5% | 22.8% | <0.001 |
| Male (%) | 152(39.9) | 231(41.8) | 171(35.3) | 119(42.0) | 0.126 |
| Weight (kg) | 61.1±8.8 | 61.4±9.3 | 60.6±9.4 | 60.8±8.7 | 0.444 |
| BMI (kg/m^2^) | 22.3±2.5 | 22.5±2.6 | 22.5±2.6 | 22.4±2.4 | 0.717 |
| SBP (mmHg) | 117±12 | 119±13 | 119±12 | 122±13 | <0.001 |
| DBP (mmHg) | 75±7 | 76±8 | 77±7 | 79±7 | <0.001 |
| ALT (U/L) | 17(12-25) | 18(14-26) | 21(15-26) | 20(16-27) | <0.001 |
| AST (U/L) | 20(17-24) | 21(18-25) | 21(19-25) | 24(20-27) | <0.001 |
| GGT (U/L) | 20(16-24) | 19(15-23) | 19(16-23) | 20(16-24) | 0.032 |
| LDH (U/L) | 163(139-186) | 162(140-186) | 164(139-186) | 159(135-183) | 0.321 |
| CHOL (mmol/L) | 5.0±0.9 | 5.3±0.8 | 5.6±0.8 | 5.4±0.9 | <0.001 |
| TG (mmol/L) | 1.18±0.55 | 1.41±0.60 | 1.29±0.56 | 1.47±0.60 | <0.001 |
| HDL-c (mmol/L) | 1.46±0.39 | 1.40±0.34 | 1.43±0.35 | 1.40±0.33 | 0.038 |
| LDL-c (mmol/L) | 3.24±0.71 | 3.40±0.76 | 3.66±0.72 | 3.41±0.81 | <0.001 |
| FBG (mmol/L) | 5.0±0.7 | 5.2±0.8 | 5.5±0.9 | 5.8±1.3 | <0.001 |
| Creatinine (μmol/L) | 70±16 | 72±16 | 70±15 | 76±17 | <0.001 |
| BUN (mmol/L) | 5.6±1.4 | 5.5±1.4 | 5.6±1.4 | 5.5±1.4 | 0.879 |
| Uric acid (μmol/L) | 318±47 | 316±52 | 331±53 | 342±53 | <0.001 |
| Platelet (*10^9^/L) | 241±40 | 243±41 | 242±40 | 244±41 | 0.717 |

Data are expressed as n (%), mean ± standard deviation and median (quartile).

Abbreviations: NAFLD, non-alcoholic fatty liver disease; BMI, body mass index; SBP, systolic blood pressure; DBP, diastolic blood pressure; ALT, alanine aminotransferase; AST, aspartate aminotransferase; GGT, gamma glutamyl transpeptidase; LDH, lactic dehydrogenase; CHOL, total cholesterol; TG, triglycerides; HDL-c, high-density lipoprotein-cholesterol; LDL-c, low-density lipoprotein-cholesterol; FBG, fasting blood glucose; BUN, blood urea nitrogen

Table S3. The univariate Cox regression analysis of risk factors for incidence of NAFLD during follow-up

| **Variable** | **ALL** | *P* | **20-34 years old** | *P* | **35-49 years old** | *P* | **50-64 years old** | *P* | **≥65 years old** | *P* |
| --- | --- | --- | --- | --- | --- | --- | --- | --- | --- | --- |
|  | HR (95%CI) |  | HR (95%CI) |  | HR (95%CI) |  | HR (95%CI) |  | HR (95%CI) |  |
| Male | 1.20(0.92-1.49) | 0.30 | 0.90(0.80-1.02) | 0.21 | 1.55(1.24-1.87) | <0.001 | 0.85(0.69-1.03) | 0.85 | 1.56(1.22-1.92) | <0.001 |
| **Baseline factors** |  |  |  |  |  |  |  |  |  |  |
| BMI (increase 1kg/m^2^) | 1.14(1.02-1.28) | <0.001 | 1.07(0.98-1.17) | 0.85 | 1.09(1.06-1.13) | <0.001 | 1.24(1.10-1.39) | <0.001 | 1.10(0.96-1.25) | 0.909 |
| SBP (increase 1mmHg) | 1.00(0.99-1.01) | 0.054 | 1.00(0.99-1.01) | 0.967 | 1.00(0.99-1.01) | 0.211 | 1.02(1.01-1.03) | <0.001 | 1.03(1.01-1.05) | <0.001 |
| DBP (increase 1mmHg) | 1.01(0.99-1.03) | 0.55 | 1.00(0.99-1.02) | 0.83 | 1.01(1.00-1.02) | 0.24 | 1.01(0.99-1.02) | 0.29 | 1.01(0.99-1.03) | 0.12 |
| ALT (increase 1U/L) | 1.01(0.99-1.02) | 0.14 | 1.01(1.00-1.02) | <0.001 | 1.01(1.00-1.02) | 0.01 | 1.00(0.99-1.01) | 0.95 | 1.02(1.01-1.02) | 0.01 |
| AST (increase 1U/L) | 1.00(0.98-1.01) | 0.50 | 1.02(1.02-1.02) | 0.01 | 1.01(1.00-1.02) | 0.04 | 1.00(0.98-1.01) | 0.64 | 1.01(0.99-1.03) | 0.20 |
| GGT (increase 1U/L) | 1.00(0.98-1.01) | 0.65 | 1.00(0.99-1.02) | 0.72 | 0.98(0.96-1.00) | 0.04 | 0.99(0.97-1.02) | 0.47 | 1.01(0.98-1.03) | 0.35 |
| LDH (increase 1U/L) | 1.00(0.99-1.00) | 0.08 | 1.00(0.99-1.00) | 0.87 | 1.00(0.99-1.00) | 0.40 | 1.00(0.99-1.00) | 0.24 | 1.00(0.99-1.00) | 0.14 |
| CHOL (increase 1mmol/L) | 1.30(0.80-1.85) | 0.65 | 1.21(1.02-1.42) | 0.01 | 1.25(1.03-1.48) | <0.001 | 1.33(1.05-1.63) | <0.001 | 1.42(1.02-1.85) | <0.001 |
| TG (increase 1mmol/L) | 1.58(0.88-2.32) | 0.20 | 1.43(0.98-2.01) | 0.12 | 1.34(0.95-1.86) | 0.09 | 1.52(1.14-2.12) | <0.001 | 1.72(1.09-2.60) | <0.001 |
| HDL-c (increase 1mmol/L) | 0.80(0.65-0.95) | <0.001 | 0.57(0.20-1.66) | 0.30 | 0.89(0.78-1.05) | 0.23 | 0.40(0.28-0.58) | <0.001 | 0.87(0.66-1.08) | 0.41 |
| LDL-c (increase 1mmol/L) | 1.45(1.28-1.65) | <0.001 | 1.26(0.86-1.85) | 0.24 | 1.71(1.05-2.65) | <0.001 | 1.39(1.15-1.68) | <0.001 | 1.83(1.10-2.90) | <0.001 |
| FBG (increase 1mmol/L) | 1.16(1.11-1.22) | <0.001 | 0.92(0.77-1.09) | 0.326 | 1.07(0.95-1.21) | 0.268 | 1.10(0.99-1.22) | 0.06 | 1.10(0.98-1.22) | 0.12 |
| Creatinine (increase 1μmol/L) | 1.00(1.00-1.00) | 0.445 | 1.00(0.99-1.00) | 0.10 | 1.00(1.00-1.00) | 0.01 | 1.00(0.99-1.01) | 0.230 | 1.00(0.99-1.01) | 0.81 |
| BUN (increase 1mmol/L) | 0.99(0.96-1.03) | 0.65 | 1.00(0.93-1.07) | 0.90 | 0.98(0.92-1.04) | 0.46 | 1.03(0.97-1.10) | 0.30 | 0.94(0.87-1.02) | 0.14 |
| Uric acid (increase 1μmol/L) | 1.00(0.99-1.00) | 0.13 | 1.01(0.97-1.06) | 0.35 | 1.01(1.00-1.02) | 0.02 | 1.01(0.99-1.03) | 0.24 | 1.04(1.01-1.07) | <0.001 |
| **Dynamic factors** |  |  |  |  |  |  |  |  |  |  |
| ΔBMI (increase 1kg/m^2^) | 1.70(1.58-1.84) | <0.001 | 2.14(2.00-2.30) | <0.001 | 1.60(1.52-1.69) | <0.001 | 1.51(1.08-1.94) | <0.001 | 1.10(0.85-1.37) | 0.30 |
| ΔSBP (increase 1mmHg) | 1.05(0.97-1.13) | 0.50 | 0.99(0.98-1.01) | 0.65 | 0.99(0.98-1.00) | 0.35 | 1.02(0.94-1.10) | 0.30 | 1.05(0.99-1.11) | 0.42 |
| ΔDBP (increase 1mmHg) | 1.00(0.98-1.01) | 0.60 | 1.01(1.00-1.02) | 0.01 | 1.01(1.00-1.02) | 0.03 | 1.00(0.98-1.01) | 0.53 | 1.02(0.99-1.04) | 0.17 |
| ΔALT (increase 1U/L) | 1.00(0.99-1.01) | 0.65 | 1.01(1.00-1.01) | <0.001 | 1.02(1.01-1.03) | <0.001 | 1.01(1.00-1.01) | <0.001 | 0.99(0.98-1.01) | 0.33 |
| ΔAST (increase 1U/L) | 1.01(0.99-1.02) | 0.06 | 1.02(1.02-1.03) | <0.001 | 1.01.00-1.02) | <0.001 | 1.01(1.00-1.03) | <0.001 | 1.01(1.00-1.02) | <0.001 |
| ΔGGT (increase 1U/L) | 1.01(1.00-1.02) | 0.01 | 1.01(0.99-1.02) | 0.44 | 1.01(0.99-1.02) | 0.40 | 1.02(1.00-1.03) | 0.01 | 1.02(1.00-1.03) | 0.12 |
| ΔLDH (increase 1U/L) | 1.00(1.00-1.01) | 0.30 | 1.00(0.99-1.00) | 0.82 | 1.00(1.00-1.01) | 0.28 | 1.00(1.00-1.01) | 0.93 | 1.00(0.99-1.00) | 0.86 |
| ΔCHOL (increase 1mmol/L) | 1.23(0.88-1.60) | 0.66 | 1.12(1.04-1.22) | <0.001 | 1.26(1.06-1.47) | <0.001 | 1.33(1.04-1.64) | <0.001 | 1.45(1.04-1.87) | <0.001 |
| ΔTG (increase 1mmol/L) | 2.20(1.54-3.04) | <0.001 | 1.85(0.78-3.02) | 0.45 | 2.12(0.87-4.34) | 0.15 | 2.23(1.10-3.40) | <0.001 | 5.54(3.85-7.97) | <0.001 |
| ΔHDL-c (increase 1mmol/L) | 0.71(0.62-0.81) | <0.001 | 0.78(0.56-1.05) | 0.15 | 0.75(0.54-1.01) | 0.07 | 0.74(0.48-1.13) | 0.56 | 0.88(0.70-1.08) | 0.33 |
| ΔLDL-c (increase 1mmol/L) | 1.05(0.97-1.13) | 0.22 | 1.34(1.03-1.76) | <0.001 | 1.17(1.05-1.30) | <0.001 | 1.20(1.08-1.65) | <0.001 | 1.21(1.02-1.43) | <0.001 |
| ΔFBG (increase 1mmol/L) | 1.20(1.10-1.31) | <0.001 | 1.20(1.09-1.33) | <0.001 | 1.15(1.03-1.28) | 0.01 | 1.16(1.06-1.27) | <0.001 | 1.38(1.25-1.53) | <0.001 |
| ΔCreatinine (increase 1μmol/L) | 1.00(1.00-1.00) | 0.09 | 1.00(0.99-1.00) | 0.99 | 1.00(1.00-1.01) | 0.06 | 1.00(1.00-1.01) | 0.22 | 1.00(1.00-1.01) | 0.75 |
| ΔBUN (increase 1mmol/L) | 1.00(0.98-1.03) | 0.60 | 1.01(0.96-1.06) | 0.80 | 1.04(0.99-1.08) | 0.10 | 0.96(0.92-1.00) | 0.07 | 1.02(0.97-1.08) | 0.48 |
| ΔUric acid (increase 1μmol/L) | 1.04(1.00-1.08) | <0.001 | 1.01(0.95-1.09) | 0.10 | 1.01(1.00-1.02) | 0.03 | 1.03(1.01-1.05) | <0.001 | 1.02(1.01-1.03) | <0.001 |

Abbreviations: NAFLD, non-alcoholic fatty liver disease; HR, hazard ratio; BMI, body mass index; SBP, systolic blood pressure; DBP, diastolic blood pressure; ALT, alanine aminotransferase; AST, aspartate aminotransferase; GGT, gamma glutamyl transpeptidase; LDH, lactic dehydrogenase; CHOL, total cholesterol; TG, triglycerides; HDL-c, high-density lipoprotein-cholesterol; LDL-c, low-density lipoprotein-cholesterol; FBG, fasting blood glucose; BUN, blood urea nitrogen.

Δ = change in variable i.e. follow-up minor bsaeline measurement.


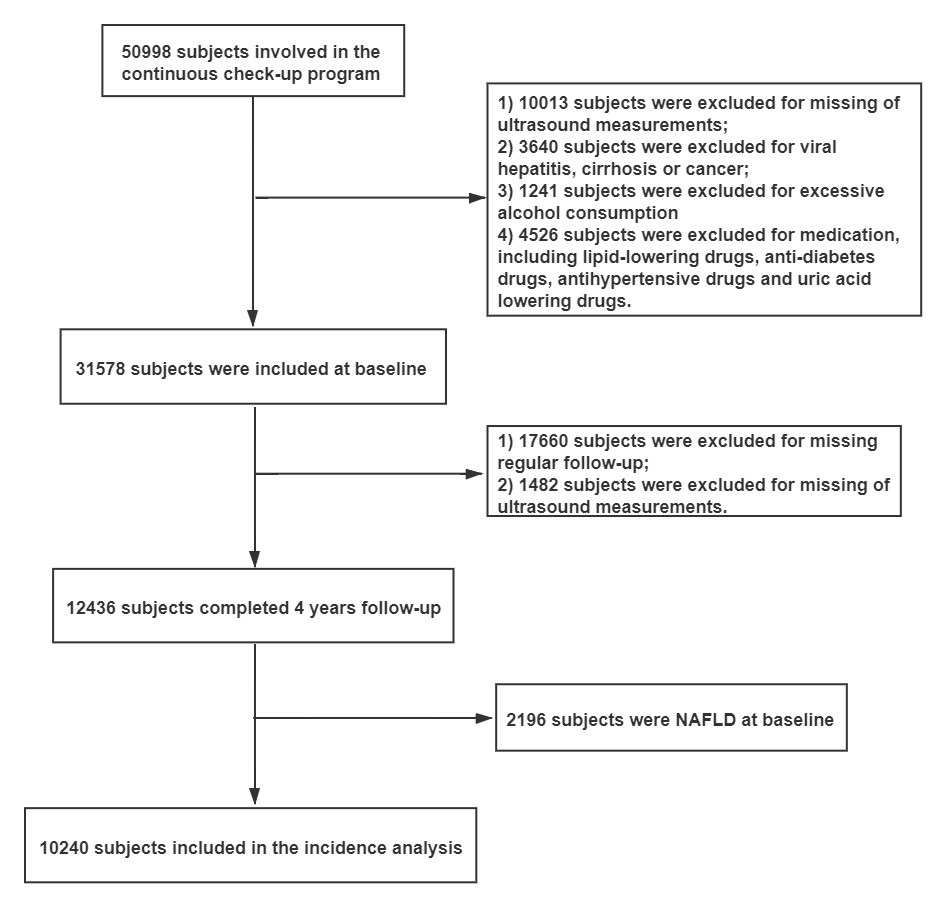


**Figure S1**. Study flow diagram for longitudinal cohort.


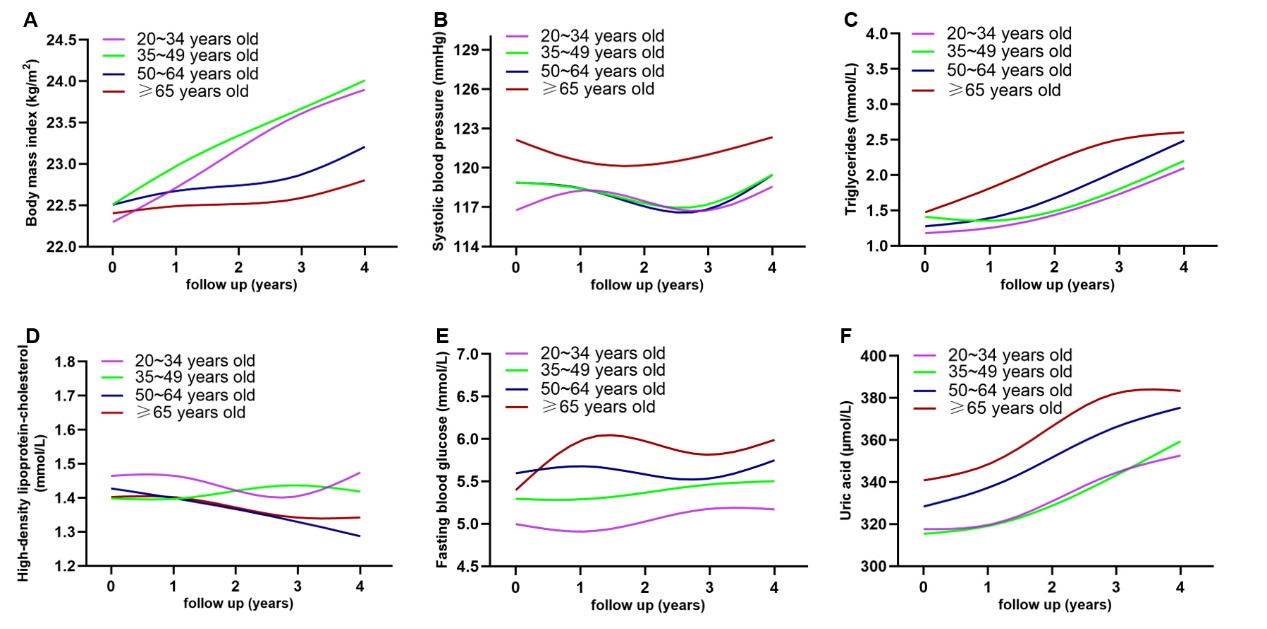


**Figure S2.** Metabolic trajectories before NAFLD onset strategied by different ages.


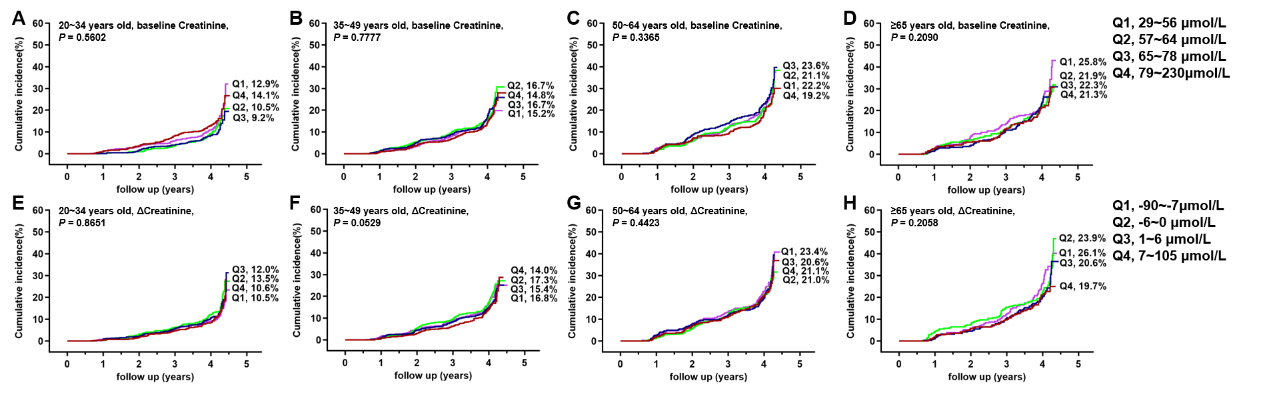


**Figure S3**. The cumulative incidence of NAFLD in the quartiles (Q) of baseline creatinine [A-D] and dynamic changes in creatinine [E-H] for newly onset NAFLD. The corresponding quartiles of the variables were shown on the right side of the picture. Because all patients with completed follow-up for 4 years, therefore the numbers of patients at risk for each K-M curves were not shown.
